# Virulence Traits and Population Genomics of the Black Yeast *Aureobasidium melanogenum*

**DOI:** 10.3390/jof7080665

**Published:** 2021-08-16

**Authors:** Anja Černoša, Xiaohuan Sun, Cene Gostinčar, Chao Fang, Nina Gunde-Cimerman, Zewei Song

**Affiliations:** 1 Department of Biology, Biotechnical Faculty, University of Ljubljana, 1000 Ljubljana, Slovenia; anja.cernosa@bf.uni-lj.si (A.Č.); nina.gunde-cimerman@bf.uni-lj.si (N.G.-C.); 2 BGI-Shenzhen, Beishan Industrial Zone, Shenzhen 518083, China; sunxiaohuan@genomics.cn (X.S.); fangchao@genomics.cn (C.F.); songzewei@genomics.cn (Z.S.); 3 Lars Bolund Institute of Regenerative Medicine, BGI-Qingdao, Qingdao 266555, China

**Keywords:** *Aureobasidium melanogenum*, virulence, population genomics, siderophores, hemolysis, hydrocarbons, neurotransmitters, diploidy

## Abstract

The black yeast-like fungus *Aureobasidium melanogenum* is an opportunistic human pathogen frequently found indoors. Its traits, potentially linked to pathogenesis, have never been systematically studied. Here, we examine 49 *A. melanogenum* strains for growth at 37 °C, siderophore production, hemolytic activity, and assimilation of hydrocarbons and human neurotransmitters and report within-species variability. All but one strain grew at 37 °C. All strains produced siderophores and showed some hemolytic activity. The largest differences between strains were observed in the assimilation of hydrocarbons and human neurotransmitters. We show for the first time that fungi from the order Dothideales can assimilate aromatic hydrocarbons. To explain the background, we sequenced the genomes of all 49 strains and identified genes putatively involved in siderophore production and hemolysis. Genomic analysis revealed a fairly structured population of *A.* *melanogenum*, raising the possibility that some phylogenetic lineages have higher virulence potential than others. Population genomics indicated that the species is strictly clonal, although more than half of the genomes were diploid. The existence of relatively heterozygous diploids in an otherwise clonal species is described for only the second time in fungi. The genomic and phenotypic data from this study should help to resolve the non-trivial taxonomy of the genus *Aureobasidium* and reduce the medical hazards of exploiting the biotechnological potential of other, non-pathogenic species of this genus.

## 1. Introduction

*Aureobasidium melanogenum* (phylum Ascomycota; class Dothideomycetes; order Dothideales) is a polyextremotolerant black yeast-like fungus of increasing medical importance [1]. *A. melanogenum* can survive various extreme conditions, from high concentrations of salt to low temperatures and extreme environments [2,3,4]. The species *A. melanogenum* was described upon its genome sequencing [5] and was previously classified as a variety of *Aureobasidium pullulans*, a ubiquitous species with numerous biotechnological uses [6]. As a consequence, these two species are often difficult and sometimes impossible to distinguish, especially in the older, but sometimes also in more recent publications, some of which continue to follow the old and outdated taxonomy.

Strains of *A. melanogenum* can be isolated from diverse, mainly oligotrophic and aqueous habitats [5]—they were found in hypersaline water [7], in Greenland glacial ice [8], but also in association with plants [9,10] and in desert soil [11]. Importantly, *A. melanogenum* can colonize indoor environments and is found often in large numbers in washing machines [12], dishwashers [13], tap water [14], house dust [15], and food [16].

The most important characteristic of *A. melanogenum* that distinguishes it from other *Aureobasidium* species is its ability to grow at human body temperature [5], a trait considered crucial for its potential to cause human opportunistic infections [17]. It is believed to be the only species of the genus with this capability [5,18]. *A. melanogenum* has been associated with various infections in immunocompromised patients, such as cutaneous, ocular, catheter-related, pulmonary, and peritoneal infections [5,19,20,21,22,23]. Cases of systemic infections have also been reported [5,24]. As noted by Gostinčar et al. [5], all pathogenic “*A. pullulans*” described to date are likely misclassified strains of *A. melanogenum*.

In addition to thermotolerance, *A. melanogenum* has other traits potentially contributing to its virulence: polymorphism, melanization, oligotrophy, and the newly discovered ability to assimilate aromatic compounds and human neurotransmitters.

As has also been reported for other species of *Aureobasidium*, *A. melanogenum* is phenotypically exceedingly plastic and can switch between two major morphologies: yeast and hyphae [4,25]. Such dimorphism is typical of several fungal pathogens: in the environment, they grow as mycelia, but when they invade the human body, they convert to yeast growth. For many pathogenic fungi, the main trigger of transformation is the change of temperature [26]. As implied by its name, *A. melanogenum* produces melanin, and it does so to a greater extent than other *Aureobasidium* species [5]. Despite some contradictory results on the importance of melanization for pathogenesis [27], melanin has numerous proposed roles in virulence and is thought to be associated with modulating the immune response (inhibiting some processes such as phagocytosis and activating others by serving as a pathogen-associated molecular pattern), protecting against oxidative stress, and increasing resistance to antifungal drugs [28,29,30].

Another trait of *A. melanogenum* with a possible role in the survival of the fungus in the human body is the production of siderophores [3,31]. Iron limitation is one of the fundamental defensive strategies of the host since iron is an essential cofactor of enzymes in many basic metabolic pathways [32]. The production of siderophores, iron-binding molecules, during infection is a widespread strategy used to overcome iron limitation in the host [32], although it primarily likely evolved for growth in out-of-host oligotrophic conditions [5,17]. The nutritional versatility of *A. melanogenum* includes the ability to assimilate hydrocarbons and use them as the sole source of carbon and energy [3]. Some studies linked the assimilation of hydrocarbons to specific patterns of neural infection [30,33]. Growth on human neurotransmitters was suggested as one of the modes of pathogenesis [30].

Finally, substantial antifungal resistance of some *A. melanogenum* strains has been reported, which could narrow the options for the treatment of infections with this pathogen. For example, some strains were resistant to fluconazole and had high MICs of voriconazole, isavuconazole, caspofungin, and micafungin [8,34].

To date, only a few genomes of *A. melanogenum* were fully sequenced. These genomes were about 26 Mbp large in the case of haploid strains and about 51 Mbp in the case of a diploid strain [5,35,36]. The GC content of genomic DNA was around 50% [5,36]. The genome assembly of the first sequenced genome of *A. melanogenum* had fewer repetitive sequences than *A. pullulans* and contained a homothallic mating-type locus, with MAT1 and MAT2 in the opposite orientation [5].

More genomes are available for the related species *A. pullulans*, where a population genomic analysis provided some insights into the biology of the species. For example, no population structuring was discovered [37], indicating that the vast phenotypic and ecological variability within the species is enabled with no obvious specialization to some of the many different habitats the species can inhabit. While the comparison of *A. melanogenum* genomes might be expected to lead to a similar conclusion due to the close relatedness of the species; closely related species sometimes substantially differ in their reproductive and adaptation strategies [38]. Information on the amount of recombination within *A. melanogenum* and possible specialization on a subspecies level would therefore be valuable, especially in light of the clinical relevance of the species and the possibility of different subpopulations having different potential for pathogenesis.

Despite some published data on traits possibly related to the virulence of *A. melanogenum*, a systematic overview of these traits on a larger selection of strains is not available. In this study, we compared the virulence-related traits of 49 *A. melanogenum* strains (Table 1) and eight other *Aureobasidium* spp. (Table 2) from different habitats and geographic locations to determine possible differences in the virulence potential between the strains and the different species. We focused on traits that have not been systematically studied previously in *A. melanogenum*: hemolysis, degradation of hydrocarbon compounds, and growth on five different neurotransmitters as the sole carbon source. We extended this dataset by sequencing and comparing the genomes of 49 strains of *A. melanogenum* (Table 1) to check whether any differences in virulence-related phenotypes reflect the population structure of the species, possibly linked to the ecological specialization of the strains.

## 2. Materials and Methods

### 2.1. Virulence Factors

#### 2.1.1. Strains and Growth Conditions

Strains of the genus *Aureobasidium* (Table 1 and Table 2) isolated from different habitats around the world were obtained from the Culture Collection Ex of the Infrastructural Centre Mycosmo (Department of Biology, Biotechnical Faculty, University of Ljubljana, Slovenia). All strains were previously identified by their internal transcribed spacer sequences. Cultures were maintained on potato dextrose agar (PDA) (Biolife, Milano, Italy).

For all tests, we prepared cell suspensions of yeast-like strains in saline to OD_600_ = 1.0. In the case of strains with filamentous growth, the media were inoculated with 4 mm diameter mycelial plugs from an actively growing colony.

#### 2.1.2. Growth at Human Body Temperature

To test for growth at human body temperature (37 °C), we spotted 5 µL of cell suspensions or placed plugs of mycelium onto defined yeast nitrogen base (YNB) medium (pH 7.0) in three replicates and incubated the plates for 7 days at 24 °C and 37 °C. YNB medium (pH 7.0) contained: 0.17% yeast nitrogen base (Qbiogene, Carlsbad, CA, USA), 0.5% ammonium sulphate (Sigma-Aldrich, Saint Louis, MO, USA), 2% glucose (Fisher Scientific, Hampton, NH, USA), and 2% agar (Formedium, Hunstanton, UK), in deionized water.

#### 2.1.3. Siderophore Production

Siderophore production was determined using the chrome azurol S (CAS) agar plate assay, as previously described by Milagres et al. [39] and Schwyn and Neilands [40]. Briefly, two solutions were prepared. The first was prepared as 10 mL of 1 mM FeCl_3_ × 6H_2_O (Sigma Aldrich, USA) in 10 mM HCl (Merck, Darmstadt, Germany), mixed with 50 mL CAS solution (Acros Organics, Geel, Belgium) and 40 mL hexadecyltrimethylammonium bromide (CTAB) (Sigma Aldrich, USA). The second was prepared as 30.24 g of piperazine N,N′ bis(2 ethanesulfonic acid) (PIPES) (Acros Organics, USA), 12 g of the 50 (*w*/*v*) NaOH (Sigma Aldrich, USA), 20 g of malt extract (Biolife, Italy), 1 g of peptone (Merck, Germany), 20 g of glucose, and 20 g of agar in 900 mL of deionized water. Both solutions were autoclaved separately and combined after cooling. Plates were inoculated with 5 µL of cell suspension or plugs of mycelium in three replicates and incubated for three weeks at 24 °C and 37 °C. When the strain produced siderophores, a yellow, orange, or pink discoloration was observed around the colony.

We expressed the relative amount of produced siderophores according to Zajc et al. [3]:

(1) $$\mathrm{Amount}\;\mathrm{of}\;\mathrm{siderophore}\;\mathrm{produced}=\;\left(\mathrm{diameter}\;\mathrm{of}\;\mathrm{colony}\;\mathrm{and}\;\mathrm{discoloration}\;\mathrm{zone}\right)\;\times \;{\left(\mathrm{diameter}\;\mathrm{of}\;\mathrm{colony}\right)}^{-1}$$

#### 2.1.4. Hemolytic Assay

To determine the hemolytic activity, 5 µL of cell suspensions or plugs of mycelium in three replicates were spotted on blood agar base plates (Biolife, Italy) with 5% sterile bovine blood (Acila, Mörfelden, Germany), according to Perini et al. [8]. Plates were incubated for 7 days at 24 °C and 37 °C. The strain was classified as alpha-hemolytic when a green zone of discoloration was observed in the medium, beta-hemolytic when a clear zone was observed indicating complete lysis of the erythrocytes of the blood agar plate, and gamma-hemolytic when no change was observed in the medium [41].

#### 2.1.5. Assimilation of Hydrocarbons

Assimilation of hydrocarbons was determined according to Satow et al. [42] and modified by Zajc et al. [3]. Liquid YNB medium (pH 7.0) was prepared without any carbon source and, after autoclaving, supplemented with 20% (*v*/*v*) mineral oil and 20% (*v*/*v*) n-hexadecane (both Sigma Aldrich, USA) as the sole carbon source. Both mineral oil and n-hexadecane were filter-sterilized before use. The test tubes were inoculated with 100 µL of cell suspension or plugs of mycelium in three replicates and incubated without shaking for one month at room temperature.

#### 2.1.6. Assimilation of Neurotransmitters

To detect the assimilation of selected neurotransmitters, we used a modified M9 minimal medium (pH 7.4) [43], consisting of 0.6% Na_2_HPO_4_ (Sigma Aldrich, USA), 0.3% KH_2_PO_4_ (Sigma-Aldrich, USA), 0.05% NaCl (Fisher Scientific, USA), 0.1% NH_4_Cl (Merck, Germany), and 0.15% agar in Milli-Q water. To the autoclaved and cooled medium were added 5 mL of 40% (*w*/*v*) glucose, and 1 mL of microelements [3 mM (NH_4_)_6_Mo_7_O_24_ × 4H_2_O (Sigma Aldrich, USA), 400 mM H_3_BO_3_ (Merck, Germany), 30 mM CoCl_2_ × 6H_2_O (Merck, Germany), 10 mM CuSO_4_ × 5H_2_O (Merck, Germany), 80 mM MnCl_2_ × 4H_2_O (Sigma Aldrich, USA), 10 mM ZnSO_4_ × 7H_2_O (Sigma Aldrich, USA), 1 mL of 1 M MgSO_4_ (Carlo Erba, Milano, Italy), 100 µL of 1 M CaCl_2_ (Gram-mol, Zagreb, Croatia), and 200 µL of 5 mM FeSO_4_ (Sigma Aldrich, USA)]. After autoclaving, we added filter-sterilized 0.1 M of one of the selected neurotransmitters: acetylcholine (ACh), γ-aminobutyric acid (GABA), glycine (Gly), glutamate (Glu), and dopamine (DA) (all Sigma-Aldrich, USA). M9 minimal medium (pH 7.4) without added neurotransmitters was used as a control. Plates were inoculated with 10 µL of cell suspension or plugs of mycelium in three replicates and incubated for one month at 24 °C and 37 °C. Assimilation was assessed by comparing growth on plates with and without added neurotransmitters.

### 2.2. Genome Sequencing and Population Genomics

#### 2.2.1. Culture, Medium and Growth Conditions

Forty-nine *A. melanogenum* strains were inoculated into a liquid YNB medium (pH 7.0) and grown at 24 °C using a rotary shaker at 180 rpm. Biomass was harvested at the mid-exponential growth phase by centrifugation (10,000× *g* for 10 min, room temperature). Pellets were frozen in liquid nitrogen and stored at −80 °C until DNA isolation.

#### 2.2.2. DNA Isolation

Biomass for DNA sequencing was homogenized with a pestle and mortar while frozen in liquid nitrogen. 100 mg of the homogenized biomass was placed in 2-mL microcentrifuge tubes with a stainless-steel ball. Samples were placed in PTFE holders pre-cooled in liquid nitrogen and then homogenized (Retsch Mixer Mill 301; ThermoFisher Scientific, USA) at 20 Hz for 1 min. The homogenates were placed on ice and used for DNA extraction with UltraClean Microbial DNA Isolation Kit (MO BIO Laboratories, Carlsbad, CA, USA) according to the manufacturer’s instructions. RNA was removed using RNAse A (ThermoFisher Scientific, USA). The amount, purity, and integrity of the isolated DNA were assessed by agarose electrophoresis, spectrophotometrically (NanoDrop, 2000; ThermoFisher Scientific, USA), and by fluorometry (Qubit; ThermoFisher Scientific, USA).

#### 2.2.3. Genome Sequencing

The genome sequencing was performed using the platform BGISEQ-T5, with 2× 150-bp sequencing mode. The sequencing libraries were constructed using MGIEasy Universal DNA Library Prep Set (BGI-Shenzhen, China) following the manufacturer’s instructions. The extracted DNA, after fragmentation and size selection, underwent end repair and A-tailing and sequencing adaptor ligation. The processed DNA was denatured and circularized to make DNA nanoballs (DNBTM) before being sequenced on DNBSEQ-T5 sequencers (BGI-Shenzhen, China) [44]. Sequencing was done in a multiplexed mode. The samples were demultiplexed, assessed by FastQC, quality-trimmed (Q > 20), and adaptor-trimmed with ‘bbduk’ (https://jgi.doe.gov/data-and-tools/bbtools/, accessed on 1 October 2020).

The raw sequencing reads were deposited into the China National GeneBank Sequence Archive (CNSA) of China National GeneBank DataBase (CNGBdb) with access number CNP0001993. Sequencing reads together with assembly and annotation data were deposited in Genbank under BioProject PRJNA721240.

#### 2.2.4. Variant Calling

Sequencing reads were mapped to the reference *A. melanogenum* genome of strain EXF-3378 (CBS 110374; GenBank AYEN00000000) [5] with ‘bwa mem’. Variants were identified as described previously [37]. Briefly, the mapped reads were sorted with Samtools 1.6 [45], deduplicated with Picard 2.10.2 and used for variant calling with the Genome Analysis Toolkit 3.8 [46], following ‘Genome Analysis Toolkit (GATK) Best Practices’ with the ploidy 2, but using the ‘hard filtering’ option with parameters ‘QD < 2.0 || FS > 20.0 || SOR > 3.0 || MQ < 50.0’. The per-nucleotide density of the reference genome coverage by sequencing reads was calculated with Samtools 1.6 [45] and plotted in R using ‘ggplot2’ [47,48].

#### 2.2.5. Assembly and Annotation

Genomes were assembled using IDBA-Hybrid 1.1.3 [49], using the published *A. melanogenum* genome EXF-3378 [5] as a reference to guide the assembly process, as previously described by Gostinčar et al. [37]. The genomes were annotated with Augustus 3.4.0 [50] with the predicted coding sequences of four *Aureobasidium* spp. [5] and predicted proteins of *A. melanogenum* [5] used as hints. The prediction parameters were first fine-tuned by training within BRAKER 2.1.5 [51] on the reference *A. melanogenum* genome with RNASeq and existing annotations [5] used as hints. The genome and annotation completeness was assessed by searching for the Benchmarking Universal Single-Copy Orthologues (BUSCOs) using the BUSCO 3 software [52] in proteomic mode and with the Dothideomycetes protein dataset of the OrthoDB [53]. 

#### 2.2.6. Variant-Based Analysis

Principal component analysis of SNP data was performed with the ‘glPca’ function from the ‘adgenet’ package [54]. Linkage disequilibrium (LD) was estimated on a dataset of biallelic SNP loci and also on a more stringently filtered dataset with at least 25% frequency of each of the two alleles. For each pair of loci, the squared correlation coefficient (r2) was calculated using ‘vcftools’ [55], r2 of pairs spanning less than 2000 nucleotides were plotted as a function of distance using ‘ggplot2’ in R [47,48].

#### 2.2.7. Phylogenetic Analyses

Benchmarking Universal Single-Copy Orthologues (BUSCOs) identified as described above and present in the predicted proteomes of *A. melanogenum* in the expected number of copies (two copies in diploid genomes, one copy in haploid genomes) were used for the reconstruction of gene phylogenies. Strains 2, 16, 18, and 38 were excluded from the analysis due to a large share of their BUSCOs predicted as single-copy instead of in the expected two copies, and strain 35 was excluded due to its large phylogenetic distance to other strains. The coding sequences of each gene were aligned using MAFFT 7.215, with the ‘--auto’ option and default parameters [56]; the alignment was optimized using Gblocks 0.91, with the options ‘−b3 = 10 − b4 = 3 − b5 = n’ [57]. Alignments longer than 200 nucleotides and with an average of at least 15 nucleotide differences between gene pairs were used for phylogeny reconstruction with PhyML 3.3.20200621 [58]. The Hasegawa–Kishino–Yano 85 nucleotide substitution model [59] was used, with the alpha parameter of the gamma distribution of substitution rate categories and the proportion of invariable sites estimated using PhyML. The resulting trees were visualized using the function ‘densiTree()’ included in the ‘phangorn’ package in R [47,60]. A majority-rule consensus tree was calculated with the ‘consensus.edges’ function of the package ‘phytools’ in R, using the default parameters [47,61].

The phylogenetic network was reconstructed from the SNP data. The dissimilarity distance matrix was calculated using the R package ‘poppr’ [62] and used to construct the phylogenetic network with the Neighbor-Net algorithm, as implemented in the R package ‘phangorn’ [47,60].

Genomic distances between the genomes of published and here sequenced genomes were calculated as recommended by Gostinčar [63], using the software Dashing 0.4.0 [64] and k-mer length 20. 

#### 2.2.8. Identification of Individual Genes

In predicted proteomes of *A. melanogenum* we searched for homologues of two non-ribosomal peptide synthetases SidC and SidD by using the adenylation domains (A domains) of *A. melanogenum* SidC and SidD genes [17] with blastp and an e value cut-off at 10–80 according to Zajc et al. [3]. 

The same strategy was used to search for hemolysin homologues. The known published sequences of hemolysins used as queries were divided into three groups. Group one contained a sequence with the GenBank accession number PWO25742 [65]. For group two, the following sequences were obtained from GenBank: RKF81808 [66], XP_012049996 [67], AFR95641 [67], KIY70151 [68], and CCU75063 [69]. The following protein sequences from GenBank were placed in group three: EKD13246 [70], KAF7574864 [65], KAF7566347 [65], KAF5700252 [71], KAF4778018 [72], TQW08525 [73], RFU72965 [74], XP_018180561 [75], OHW96986 [76], KZL65578 [77], and XP_007290459 [70]. 

## 3. Results

### 3.1. Growth at Human Body Temperature

**Figure 1:**
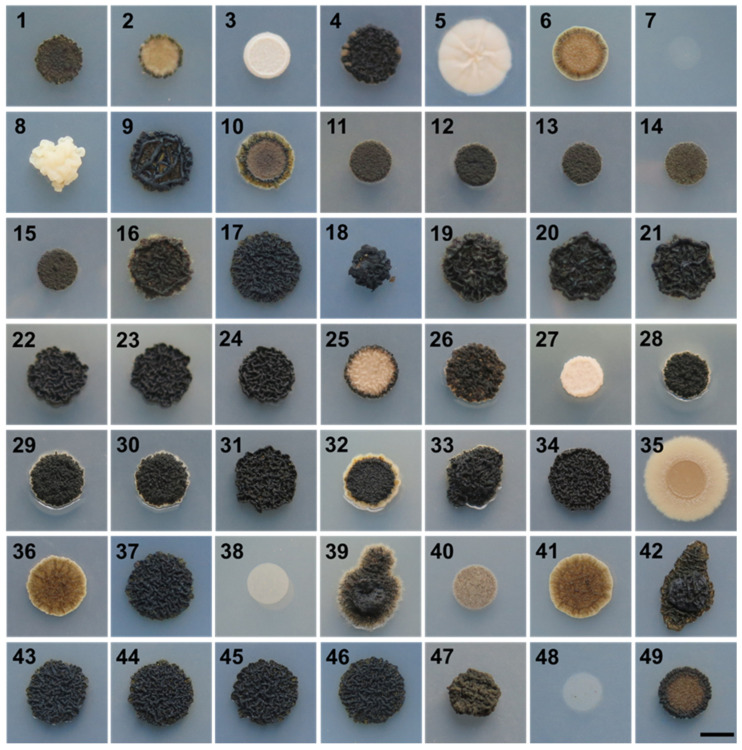
Growth of *A. melanogenum* strains on YNB medium at 37 °C. Strain numbers (1–49) are listed in Table 1. Scale bar represents 0.5 cm.

All *A. melanogenum* strains grew at 37 °C except for strain 7 (Figure 1), whereas *A. pullulans*, *A. subglaciale*, and *A. namibiae* did not grow at this temperature (Table S1).

### 3.2. Siderophore Production

All strains used in this study produced siderophores on CAS agar at 24 °C, except for one *A. pullulans* strain (EXF-3670) (Table S1). The lowest amount of siderophores was produced by *A. melanogenum* strain 27 and the highest amount by *A. melanogenum* strain 2.

When incubated at 37 °C, all strains that were able to grow also produced siderophores. The lowest amount of siderophores at this temperature were produced by *A. melanogenum* strain 39 and the highest amount by *A. melanogenum* strain 8.

**Figure 2:**
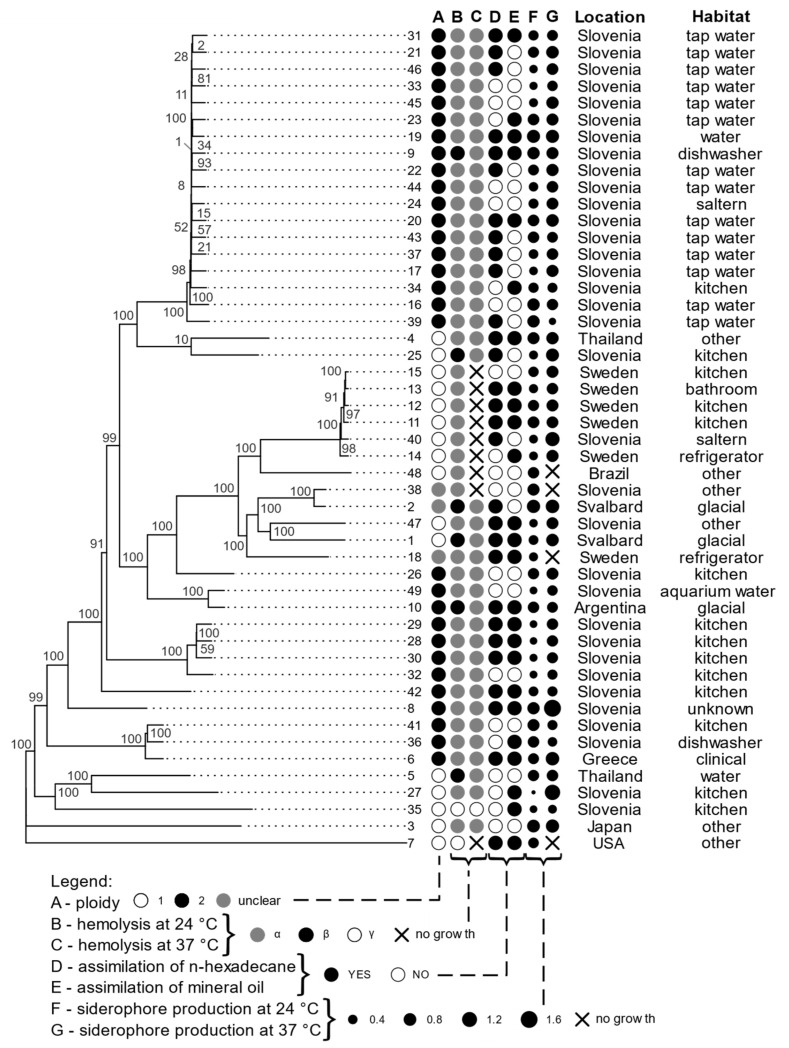
Phylogenomic tree of *A. melanogenum* strains based on whole-genome SNP data, with a selection of phenotypic traits, isolation location, and habitat. Column A of circles represents ploidy, B and C represent hemolysis at 24 °C and 37 °C, D and E show assimilation of n hexadecane and mineral oil, F and G show siderophore production at 24 °C and 37 °C (the size of the dots corresponds to the logarithmic scale of siderophore production). The ploidy of strains with genome sizes between the expected haploid and diploid sizes was marked as “unclear”.

The amount of siderophore production is represented in Figure 2 (columns F and G) and Table S1.

In all *A. melanogenum* genomes sequenced in this study, we found non-ribosomal protein synthase (NRPS) genes with adenylation domains (A domains) similar to those of SidC and SidD, NRPses responsible for the synthesis of the siderophores triacetylfusarinin and ferricrocin (Table S4). A total of 19 strains had one copy for SidC protein, 14 strains had two copies, and 16 strains had three copies of SidC protein. In total, 17 strains had one copy for SidD protein, and 32 strains had two copies. Considering diploidy, three strains had 0.5 copies for SidC protein, 30 strains had one copy, and 16 strains had 1.5 copies for SidC protein. Three strains had 0.5 copies for SidD protein, 44 had one copy, and 2 had two copies.

The highest number of copies of both proteins was five, which 16 strains had. The lowest number was two, which 15 strains had. The strain with the greatest production of siderophores at 24 °C is strain 2, which had one copy of SidC protein and two copies of SidD protein. Strain 8 had the greatest siderophore production at 37 °C and had two copies of SidC protein and two copies of SidD protein.

### 3.3. Hemolytic Assay

As presented in Figure 2 (columns B and C), the majority (84%) of *A. melanogenum* strains had an alpha-hemolytic phenotype when incubated at 24 °C. The beta-hemolytic activity was present in 12% of the strains, but only at 24 °C (strains 1, 2, 5, 9, 10, and 25). In total, 4% of strains showed gamma hemolytic activity. 

When incubated at 37 °C, the majority of *A. melanogenum* strains were still alpha-hemolytic (80%), while some strains did not grow on blood agar at this temperature at all (18%)—these were all alpha-hemolytic at 24 °C. Gamma hemolytic activity at 37 °C was present in one strain. No beta-hemolysis was observed at this temperature.

Strains of tested non-*A. melanogenum* species of the genus *Aureobasidium* were all alpha-hemolytic at 24 °C and did not grow at 37 °C (Table S1).

Searching the genomes of *A. melanogenum* for possible hemolysin homologues (Table S5), we found that four strains had no homologues corresponding to the first group of hemolysins (three of these strains were diploid), 31 strains had one copy, of which 16 were diploid, and another 14 diploid strains had two copies. In the second group of hemolysins, 27 strains had one copy, of which 11 strains were diploid, 20 had two copies, and two strains contained three copies, all diploid. In the third group of hemolysins, one strain had one copy, 18 strains had two copies, of which three strains were diploid, one had three copies, 26 had four copies, and three strains had six copies of the corresponding gene, of which all were diploid. 

Strain 35, which showed no hemolytic activity, had the fewest hemolysin homologues overall (only two—one from the second group and one from the third group). Strains 6, 36, and 41 had a total of 10 homologues, which is the highest number of homologues, but none of these strains showed beta-hemolytic activity.

### 3.4. Assimilation of Hydrocarbons

Strains of *A. melanogenum* substantially differed in their ability to utilize two selected hydrocarbons as a sole carbon source, as shown in Figure 2 (columns D in E). In total, 20% of *A. melanogenum* strains were able to assimilate only n-hexadecane, 12% were able to assimilate only mineral oil, and 39% could assimilate both n-hexadecane and mineral oil. 29% of *A. melanogenum* strains did not assimilate any of these compounds.

Other *Aureobasidium* spp. did not grow on either n-hexadecane or mineral oil, except for *A. namibiae* (strain EXF-3398) and one strain of *A. pullulans* (EXF-3670), which grew on both hydrocarbons (Table S2).

### 3.5. Assimilation of Neurotransmitters

Strains of *A. melanogenum* showed different abilities to assimilate different neurotransmitters (acetylcholine (Ach), γ-aminobutyric acid (GABA), glycine (Gly), glutamate (Glu), or dopamine (DA)). We compared colonies growing on medium with added specific neurotransmitters to medium without added neurotransmitters.

**Figure 3:**
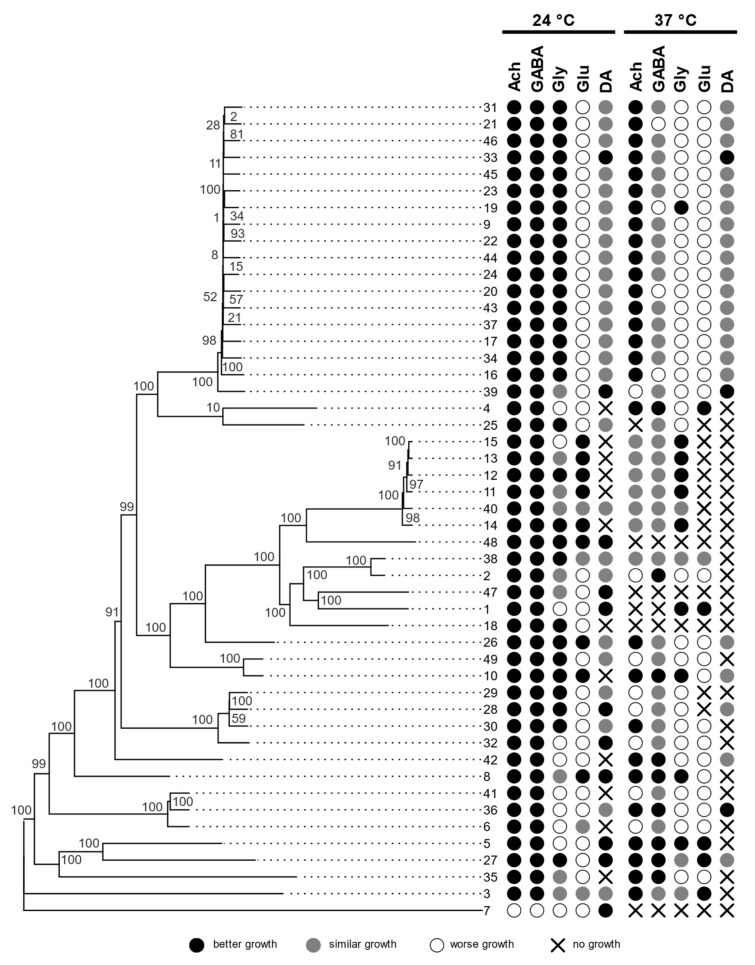
Phylogenomic tree of *A. melanogenum* strains based on whole-genome SNP data, with growth characteristics on media with different neurotransmitters, compared to the control medium with no added neurotransmitters. Assimilation of hydrocarbons is shown in the circles (acetylcholine—ACh, γ-aminobutyric acid—GABA, glycine—Gly, glutamate—Glu, dopamine—DA).

The differences were not only at the status of growth/non-growth but also in phenotype. In some strains, the neurotransmitters stimulated growth (colonies were larger compared to control), increased colony density, and/or resulted in darker pigmentation. Some strains showed all of these changes, others only one or two. In some cases, filamentous growth was triggered, which was not seen in the control. All these changes were classified as “better growth” than in the control. On the other hand, some changes were interpreted as “worse growth”, such as smaller or thinner colonies and lighter pigmentation. The results are shown in Figure 3.

While no growth was observed at 37 °C in the case of *Aureobasidium* spp. strains other than *A. melanogenum*, most of them grew on media with neurotransmitters at 24 °C, but the addition of neurotransmitters resulted in a variety of outcomes (Table S3). *A. subglaciale* strain EXF-2481 grew better on medium containing acetylcholine, γ-aminobutyric acid, glutamate, and dopamine. In addition, this strain did not grow on dopamine. Two other *A. subglaciale* strains, EXF-4631 and EXF-12298, grew better on medium containing acetylcholine and glutamate, but EXF-12298 also grew better on γ-aminobutyric acid and glycine. On dopamine, EXF-12298 grew, but EXF-4632 did not.

All *A. pullulans* strains grew better on acetylcholine and γ-aminobutyric acid, but glycine had no observable effect on growth. When growing on glutamate, EXF-150 and EXF-3670 grew better than on the control, and EXF-10629 and EXF-11318 grew similarly to the control. Only EXF-10629 failed to grow on dopamine.

*A. namibiae* (EXF-3398) grew better on all neurotransmitters compared to growth on the control medium.

### 3.6. Genomics

**Figure 4:**
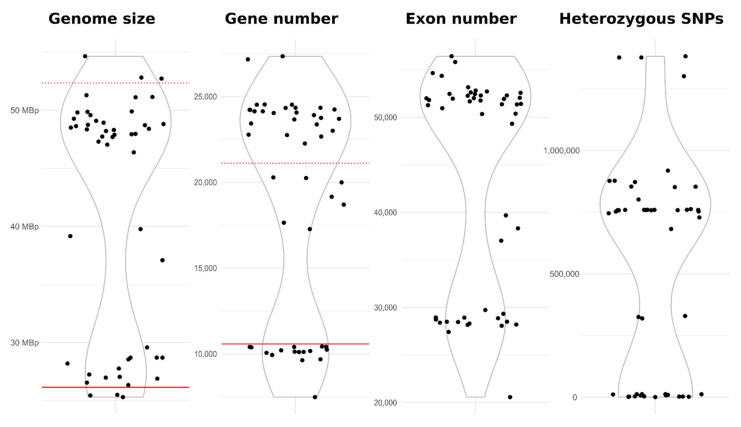
Distribution of genomes based on their size, number of predicted genes, number of exons, and number of heterozygous single nucleotide polymorphisms. The solid horizontal red line marks the value of the (haploid) reference genome, and the dashed horizontal red line marks the double of the reference value.

The average size of 49 *A. melanogenum* genomes sequenced in this study was 41.43 Mbp (Table 3) but with a bimodal distribution (Figure 4) and large differences between strains (SD 10.25 Mbp), which was also reflected in the total length of the coding sequences (22.01 Mbp ± 5.52 SD), the number of gene models (18745 ± 6405 SD), and the number of exons (43523 ± 11369 SD). The average GC content was 50.04% (SD 0.46%).

These results indicated that the genomes of most *A. melanogenum* strains are diploid (Figure 4). The genomes of 30 strains were approximately twice (average 1.89× ± SD 0.07) the size of the reference haploid genome, 16 genomes were close to the expected haploid size (average 1.04× ± SD 0.05), and the genomes of 3 strains were between haploid and diploid sizes (1.42× to 1.52× the haploid size).

**Figure 5:**
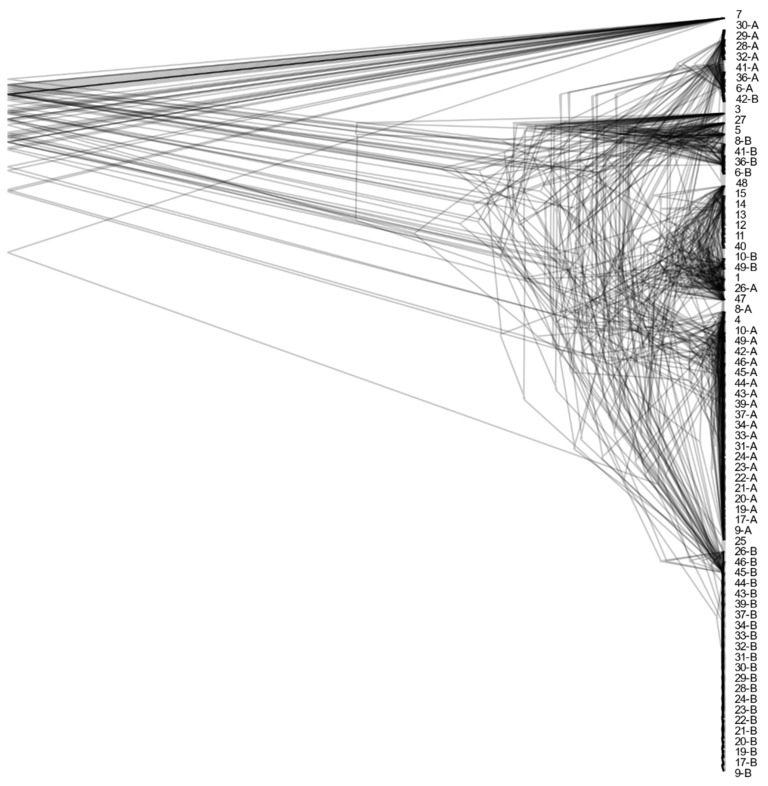
Phylogeny of *A. melanogenum* strains. Overlay of gene trees of 44 Benchmarking Universal Single-Copy Orthologs (BUSCOs). The trees were estimated by PhyML 3.1 using the Hasegawa–Kishino–Yano 85 nucleotide substitution model and estimating the alpha parameter of the gamma distribution of the substitution rate categories and the proportion of invariable sites. The numbers in the tree represent the source genomes (1–49) as numbered in this study. Duplicate genes from the same genome are marked with letters »A« and »B«.

**Figure 6:**
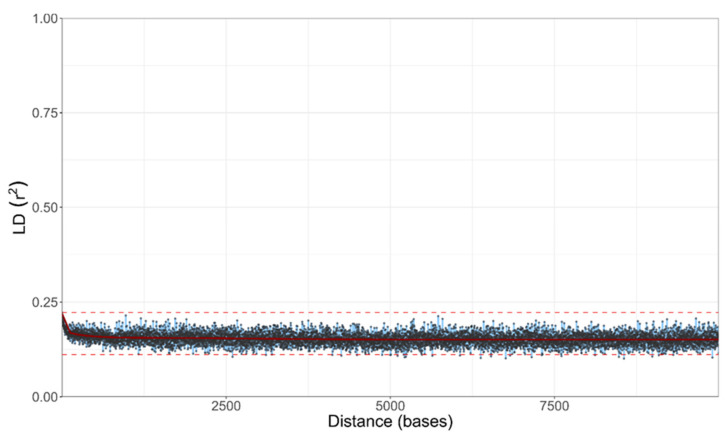
LD decay in *A. melanogenum* estimated on all biallelic loci, which were present in 25–75% of the sequenced genomes. LD measures were averaged in three nucleotide windows. Squared correlation coefficient (r2) between pairs of SNP loci plotted against the physical distance of the loci in the genome. Horizontal lines mark the maximum observed value and half of the maximum observed value. Vertical lines mark the interval of the physical distance in which the maximum value is halved.

Despite the existence of haploid and diploid strains in the population of *A. melanogenum*, no indication of sexual reproduction within the species was found. The trees of BUSCOs were topologically similar (Figure 5 and Figure S1), pointing to the lack of recombination between groups of strains. However, they showed that diploid strains are highly heterozygous hybrids of phylogenetically relatively distant haploids and that they are a result of several hybridization events. Furthermore, the linkage disequilibrium squared correlation coefficient showed no clear sign of decay even over large genomic distances, again pointing to the lack of recombination within the species (Figure 6).

**Figure 7:**
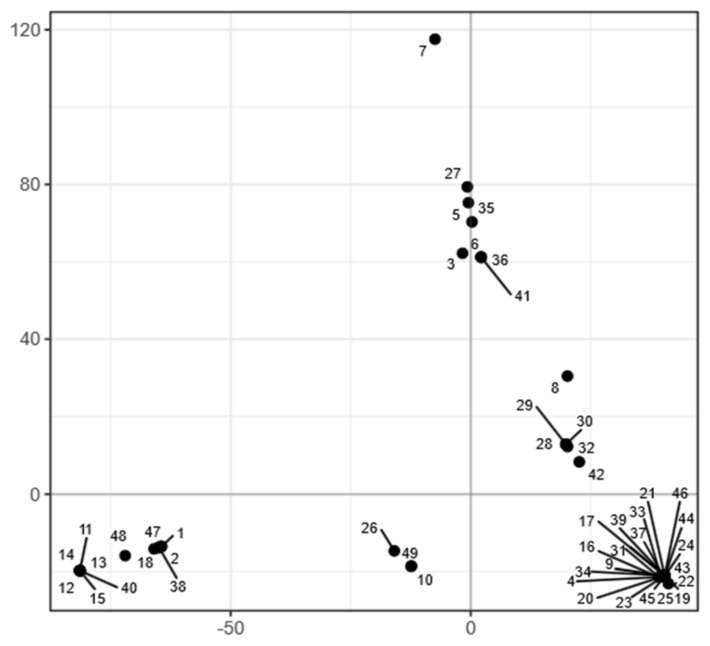
Clustering of *A. melanogenum* genomes. Principal component analysis of SNP data estimated by comparing the genomes to the reference *A. melanogenum* genome (EXF-3378). The genomes are represented by circles. The axes explain 33.11% (horizontal) and 19.85% (vertical) of variation.

In line with the lack of recombination between the strains, the population of *A. melanogenum* appeared relatively structured. PCA analysis of single nucleotide polymorphisms (SNPs) showed that the genomes of the *A. melanogenum* strains form five clusters (Figure 7). However, no connection between the clusters and habitat or sampling location was observed (Figures S2 and S3).

Strain 7 remained outside of these clusters and also had a large genomic distance to other strains (Figure S4). The dashing distance to other *A. melanogenum* strains measured at k-mer 20 was below 0.05, more than four times lower than the species threshold proposed by Gostinčar [63]. This strain thus likely belongs to a species other than *A. melanogenum*. This was confirmed by phylogenetic analyses, which again showed a large distance between strain 7 and other sequenced strains.

## 4. Discussion

*Aureobasidium melanogenum* is a black yeast-like fungus that can be found in many different extreme environments, including those in human dwellings. Our frequent contact with this species is of particular concern due to its potential for opportunistic pathogenicity [1]. It is therefore important to characterize its potential virulence factors—also in the context of substantial variability within the species. In this study, we investigated several virulence factors of *A. melanogenum*, including the growth at human body temperature, production of siderophores, hemolytic activity, and the ability to assimilate aromatic compounds and various human neurotransmitters. We also used genome sequencing and population genomics to study the population characteristics of *A. melanogenum* and compared the distribution of important virulence factors within the population.

Among the 49 strains of *A. melanogenum* used in this study, we observed great diversity at the morphological level when the strains were grown at human body temperature. The most important differences were in the speed of growth and melanization. Thermotolerance is a key virulence factor and necessary for human pathogenesis, but also a trait that most fungi lack [17,78]. The ability of all *A. melanogenum* strains to grow at 37 °C underline the human pathogenic potential of this species. Other species from the genus *Aureobasidium* did not grow at human body temperature, additionally confirming that these species may be regarded as non-pathogenic.

Unlike the different ability to grow at high temperature, some other investigated traits were widely distributed through the whole *Aureobasidium* genus. While these traits may have a role in virulence, they likely developed as a response to selection pressures outside of the host [5,17]. One such trait is the production of siderophores. Iron acquisition during infection is an important process since iron is a crucial nutrient for most organisms, and iron limitation is a fundamental and widespread immune response, but iron scarcity is common in the out-of-host environment as well. The production of siderophores by *A. melanogenum* has been intensively studied in a marine-isolated strain HN6.2 [79,80,81], which can produce four different siderophores: cyclic and linear fusarinine C (also called fusigen), ferricrocin, and hydrocyferricrocin [36,81]. The mechanisms for iron acquisition are diverse but not yet fully understood [36]. All but one of our tested *Aureobasidium* spp. strains produced siderophores at 24 °C, and *A. melanogenum* also produced them at 37 °C. However, there were differences in the number of siderophores produced. In some cases, the siderophore production changed with the temperature, but the change was not consistent: in some strains, the production increased at high temperature, while the opposite was true for others. Whether an increased production at 37 °C in some strains increases the pathogenic potential of these strains is unknown. Our in silico analyses were also consistent with the experimental results, as all tested *A. melanogenum* strains had at least one homologue of the SidC and SidD proteins, which are involved in the acquisition of iron by siderophores.

One of the strategies for iron acquisition for pathogens is the lysis of host cells and the resulting release of intracellular iron. Furthermore, cases of fungemia were reported for *A. melanogenum* [21,22,24]. Determination of hemolytic activity is a standard method for screening microbial cytolytic capacity. Screening of hemolytic activity in *A. melanogenum* showed great similarity between tested strains. The great majority of strains showed alpha-hemolysis at 24 °C and 37 °C. However, the full beta-hemolysis was observed only in six strains at 24 °C, three diploid and three haploid; three of them were isolated from glacial ice, and two were isolated from kitchens, but the clinical isolate 6 was not among them. This substantially expands on previous reports on the hemolytic activity of *A. melanogenum* [8,82], which involved only a handful of strains. Some fungi are known to produce hemolysins, proteins capable of lysing red blood cells [83]; however, nothing is known about the mechanism of hemolysis in *A. melanogenum*. The search for hemolysin homologues showed that all strains had homologues for at least one type of hemolysin. In silico analysis corresponded to some of the experimental results, e.g., strain 35, which showed no hemolytic activity, also had the fewest hemolysin homologues. However, strains 6, 36, and 41 with the highest number of hemolysin homologues showed only alpha-hemolytic activity. Overall, no correlation was observed between the number and type of hemolysin homologues and hemolytic activity, which may indicate that *A. melanogenum* produces other types of hemolysins. The expression of the genes and activity of proteins also likely play a role at least as important as the number of gene copies.

While strains of *A. melanogenum* in this analysis were relatively similar in their hemolysis profiles and production of siderophores, they differed substantially in their capacity for the assimilation of hydrocarbons. Some strains assimilated only n-hexadecane (ten strains), others only mineral oil (five strains), most assimilated both (19 strains, including the clinical isolate 6), and others none (15 strains). The reason that some strains grew only on one hydrocarbon could be due to their chemical difference: n-hexadecane is a short-chain molecule with 16 carbon atoms, while mineral oil is a complex mixture of long-chain hydrocarbons and aromatic hydrocarbons. Previous studies showed that some black yeasts from the order Chaetothyriales associated with human pathogenesis could assimilate hydrocarbons, and due to this correlation, the trait was suggested as a possible virulence factor and explained by the common chemical nature of alkylbenzenes and their metabolites and some neurotransmitters [33,84]. So far, the common opinion was that only black fungi from the order Chaetothyriales could assimilate aromatic hydrocarbons [33]. However, our results showed that more than half of *A. melanogenum* strains can assimilate aromatic hydrocarbons as well, which is the first time this trait has been demonstrated in the order Dothideales. Large variability in the of hydrocarbons in *A. melanogenum* is thus of interest, but whether it can be linked to different virulence of individual strains, for now, remains unknown. The ability to degrade hydrocarbons is notable for one more reason: it may be linked to a higher capacity for biodegradation of plastics [85]. This is particularly important since some species of the genus *Aureobasidium* have been identified as frequent colonizers of synthetic polymers and degraders of plastics [86,87]. Their genomes contain several genes with a possible role in the degradation of these compounds [5].

There are several case reports in the literature of species from the genus *Aureobasidium* causing meningitis [88,89,90], including one attributed to *A. pullulans* [24]. Many if not all of these pathogens were, in fact, likely *A. melanogenum* since this species was only described in 2014. Given their ability to occasionally cause neurological infections, we investigated the neurotropic potential of *A. melanogenum* strains by growing them on human neurotransmitters (acetylcholine, γ-aminobutyric acid, glycine, glutamate, and dopamine). Most tested *A. melanogenum* strains showed good growth but also great variability among strains. In most cases, growth on acetylcholine or γ-aminobutyric acid was better at 24 °C, while growth at 37 °C is more important for human pathogenesis. Better growth, in this case, was observed only in some strains and mostly when growing on acetylcholine. As in the case of hydrocarbon assimilation, the ability to grow on human neurotransmitters, especially at 37 °C, is not a universal trait of *A. melanogenum*; thus, we cannot speculate whether this variability is correlated with different pathogenic potentials.

When the first two genomes of *A. melanogenum* were sequenced [5,36], the genomic characteristics of *A. melanogenum* appeared very similar to those of the closely related *A. pullulans*: they were of similar size, contained a similar number of predicted genes, and were haploid. However, after sequencing additional strains, we can now observe striking differences between the two species. The study of *A. pullulans* detected a very high level of recombination within the species and showed that all of the 50 sequenced genomes were haploid [37]. In contrast, already the third published genome of *A. melanogenum* appeared to be diploid [35]. The above-presented data show that the reported diploid genome was not an outlier—in our study, the majority of sequenced strains were diploid, 33 out of 49 sequenced (Figure 5). To add to this surprising observation, other results—particularly the lack of decay of linkage disequilibrium and a high degree of congruence between phylogenies of core genes—indicated a lack of recombination within the species. This situation is nearly identical to that observed in *Hortaea werneckii*, another black yeast from Dothideomycetes, where the genome sequencing provided evidence for the presence of persistent haploid as well as diploid strains in nature. This was suggested to be a consequence of occasional hybridization between relatively heterozygous haploids, while the species was otherwise limited to clonal reproduction. Observation of a similar phenomenon in *A. melanogenum* (but not in the closely related *A. pullulans*) could mean that such reproduction strategy is more common than originally thought and should be investigated further. The existence of three *A. melanogenum* strains with the genome size between haploid and diploid might be an assembly artefact due to the lower heterozygosity of these strains, a result of the most similar parts of both haploid subgenomes being collapsed into a “haploid” sequence and the more heterozygous parts of the subgenomes correctly assembled separately as “diploid”. Alternatively, these strains could be aneuploid [91].

As shown in Figure 7, most strains group into five clusters. Strain 7 remained apart from other strains both in phylogenetic analyses and PCA of single nucleotide polymorphisms. The genomic distance between strain 7 and other *A. melanogenum* is large enough to suspect this strain belongs to a different species according to the proposed genomic criteria for delineation of fungal species [63]. According to these same criteria, strain 7 may be placed within the species *A. namibiae*. This is notable since, to date, only one strain of *A. namibiae* has been isolated [5]. However, when comparing the here investigated phenotypes of the only known *A. namibiae* strain and strain 7, these two strains differed in a large number of traits, warranting further taxonomic investigation. 

In conclusion, *A. melanogenum* is a versatile species, which can occur in many different habitats, including domestic environments, where it is expected to come in contact with humans on a regular basis. This is of particular importance due to its ability to cause occasional human opportunistic infections. Our results show numerous differences between individual *A. melanogenum* strains in traits thought to be important for virulence. It is, therefore, possible that some strains have a greater potential for opportunistic infections. The existence of stable and highly heterozygous *A. melanogenum* diploids in an otherwise clonal species is an unusual reproductive strategy described only for the second time in fungi. The genomic and phenotypic data produced by this study should help clarify the complex taxonomy of the *Aureobasidium* spp. and minimize the hazards of exploitation of the great biotechnological potential of the genus.

## Figures and Tables

**Table 1:** List of *A. melanogenum* strains used in this study.

Culture Collection Strain Number	Present Study Number	Isolation Habitat	Sampling Site Location
EXF-924	1	Glacial: ponds on sea ice	Arctic; Svalbard, Ny Alesund
EXF-926	2	Glacial: surface glacial ice	Arctic; Svalbard, Ny Alesund
EXF-3233	3	Other: deep sea (4500 m b.s.l.)	Japan
EXF-3371	4	Other: soil	Thailand
EXF-3378	5	Water: public fountain	Thailand, Bangkok
EXF-3397	6	Clinical: endoperitoneal fluid	Greece, Athens
EXF-3399	7	Other: decomposing military textile	USA, Florida
EXF-4450	8	Unknown: Iskra factory	Slovenia
EXF-5590	9	Dishwasher: rubber seal	Slovenia
EXF-6171	10	Glacial: black glacier	Argentina
EXF-7932	11	Kitchen: metal drain on the kitchen sink	Sweden
EXF-7946	12	Kitchen: metal holder for washed dishes	Sweden
EXF-8016	13	Bathroom: between faucet and sink	Sweden
EXF-8022	14	Refrigerator: inner surface	Sweden
EXF-8044	15	Kitchen: metal holder for washed dishes	Sweden
EXF-8258	16	Water: water from well	Slovenia
EXF-9877	17	Tap water	Slovenia, Rodica
EXF-11403	18	Refrigerator	Sweden
EXF-8492	19	Water: water from well	Slovenia
EXF-8678	20	Water: water from well	Slovenia, Šentvid
EXF-8689	21	Water: water from well	Slovenia, Kleče
EXF-8695	22	Water: water from well	Slovenia, Hrastje
EXF-8702	23	Water: water from well	Slovenia, Brest
EXF-8986	24	Saltern: fango mud from Sečovlje salterns	Slovenia, Sečovlje
EXF-9262	25	Kitchen: rubber on kitchen drain	Slovenia, Gomilsko
EXF-9470	26	Kitchen: counter above dishwasher	Slovenia, Gomilsko
EXF-9272	27	Kitchen: strainer basket	Slovenia, Vojnik
EXF-9298	28	Kitchen: plastic mesh on kitchen drain	Slovenia, Podlog v Savinjski dolini
EXF-9304	29	Kitchen: strainer basket	Slovenia, Podlog v Savinjski dolini
EXF-9313	30	Kitchen: sink	Slovenia, Podlog v Savinjski dolini
EXF-9454	31	Tap water	Slovenia, Podlog v Savinjski dolini
EXF-9484	32	Kitchen: counter above dishwasher	Slovenia, Velenje
EXF-9887	33	Tap water	Slovenia, Velenje
EXF-9516	34	Kitchen: sink drain	Slovenia, Zgornji Dolič
EXF-9539	35	Kitchen: strainer basket	Slovenia, Lokovica
EXF-9540	36	Dishwasher door	Slovenia, Celje
EXF-10064	37	Tap water	Slovenia, Ormož
EXF-11060	38	Other: ceiling	Slovenia, Celje
EXF-9875	39	Tap water	Slovenia, Rodica
EXF-9906	40	Saltern: *Arthrocnemum* sp. plant from Sečovlje saltern	Slovenia, Sečovlje
EXF-9911	41	Kitchen: sink drain	Slovenia, Ormož
EXF-9937	42	Kitchen: rubber on kitchen drain	Slovenia, Ljutomer
EXF-10061	43	Tap water	Slovenia, Trebnje
EXF-10062	44	Tap water	Slovenia, Litija
EXF-10066	45	Tap water	Slovenia, Planina pri Sevnici
EXF-10333	46	Tap water	Slovenia, Ljubljana
EXF-10372	47	Other: air in National Gallery restoration centre	Slovenia, Ljubljana
EXF-10726	48	Other: integument of a male alate ant of *Atta sexdens rubropilosa*	Brazil, Sao Paolo
EXF-11028	49	Aquarium water: *Proteus anguinus*	Slovenia, Ljubljana

**Table 2:** List of *Aureobasidium* spp. strains used in this study.

Culture Collection Strain Number	Genus	Isolation Habitat	Sampling Site Location
EXF-2481	*Aureobasidium subglaciale*	Glacial: subglacial ice from seawater	Arctic; Svalbard, Ny Alesund
EXF-4632	*Aureobasidium subglaciale*	Plant: decaying leaves of Convallaria sp.	Slovenia
EXF-12298	*Aureobasidium subglaciale*	Refrigerator	Sweden
EXF-150	*Aureobasidium pullulans*	Saltern: hypersaline water, active saltpans Seča, Droga Portorož	Slovenia, Portorož
EXF-3670	*Aureobasidium pullulans*	Glacial: ice at the edge of the glacier	Arctic; Svalbard, Ny Alesund
EXF-10629	*Aureobasidium pullulans*	Other: car reservoir fuel	Slovenia
EXF-11318	*Aureobasidium pullulans*	Plant: apple surface	Slovenia, Horjul
EXF-3398	*Aureobasidium namibiae*	Other: dolomitic marble	Namibia, Namib desert

**Table 3:** Statistics for the sequenced *A. melanogenum* genomes.

	Minimum	Mean	Maximum	Standard Deviation
**Genome assembly size (Mbp)**	25.31	41.43	54.65	10.25
**GC content (%)**	49.14	50.04	51.79	0.46
**CDS total length (Mbp)**	12.41	22.01	30.21	5.52
**CDS total length (% of genome)**	46.00	53.18	57.62	2.47
**Gene models (n)**	7480	18745	27345	6405
**Gene average length (bp)**	1109	1378	1945	232
**Number of exons (n)**	20572	43523	56418	11369
**Exons per gene (average)**	2.05	2.42	2.90	0.30
**Number of introns (n)**	7480	18745	27345	6405
**Intron average length (bp)**	86	94	162	11

## Data Availability

The raw sequencing reads are available in the China National GeneBank Sequence Archive (CNSA) of China National GeneBank DataBase (CNGBdb) under access number CNP0001993. Sequencing reads together with assembly and annotation data are available in Genbank under BioProject PRJNA721240.

## Supplementary Materials

The following are available online at https://www.mdpi.com/article/10.3390/jof7080665/s1, Figure S1: Majority rule consensus tree of *A. melanogenum* Benchmarking Universal Single-Copy Orthologs (BUSCOs), Figure S2: Phylogenetic network of sequenced *A. melanogenum* strains, reconstructed with the Neighbor-Net algorithm based on the dissimilarity distance matrix calculated from the SNP data, Figure S3: Clustering of *A. melanogenum* genomes (habitat), Figure S4: Clustering of *A. melanogenum* genomes (sampling location), Table S1: Growth of selected *Aureobasidium* strains on YNB medium, hemolytic activity and siderophore production at 24 °C and 37 °C, Table S2: Assimilation of hydrocarbons (n-hexadecane and mineral oil) of selected *Aureobasidium* species, Table S3: Assimilation of neurotransmitters (acetylcholine—ACh, γ-aminobutyric acid—GABA, glycine—Gly, glutamate—Glu, dopamine—DA) of selected *Aureobasidium* species at 24 °C and 37 °C, Table S4: Found non-ribosomal protein synthase (NRPS) genes with adenylation domains (A domains) similar to those of genes encoding proteins SidC and SidD in *A. melanogenum* genomes, Table S5: Found hemolysin homologues from three different groups in *A. melanogenum* genome.
