# Supplementary material for: Virulence Traits and Population Genomics of the Black Yeast Aureobasidium melanogenum
Source: J Fungi (Basel). 2021 Aug 16;7(8):665. doi: 10.3390/jof7080665 (PMC8401163; doi:10.3390/jof7080665)
Supplement: Supplementary file 1 [file jof-07-00665-s001.zip › jof-1291805-supplementary.pdf]

Table S1: Growth of selected *Aureobasidium* strains on YNB medium, hemolytic activiy and siderophore production at 24 °C and 37 °C.

	growth on YNB		hemolytic activity		siderophore production	
**Culture collection strain number**	**24 °C**	**37 °C**	**24 °C**	**37 °C**	**24 °C**	**37 °C**
**EXF-2481**	+	NG	α	NG	1,15	NG
**EXF-4632**	+	NG	α	NG	2,41	NG
**EXF-12298**	+	NG	α	NG	1,28	NG
**EXF-150**	+	NG	α	NG	1,67	NG
**EXF-3670**	+	NG	α	NG	-	NG
**EXF-10629**	+	NG	α	NG	1,20	NG
**EXF-11318**	+	NG	α	NG	1,16	NG
**EXF-5629**	+	NG	α	NG	1,26	NG
**EXF-3398**	+	NG	α	NG	2,11	NG

Legend: + growth, - no production, NG – no growth.

Table S2: Assimilation of hydrocarbons (n-hexadecane and mineral oil) of selected *Aureobasidium* species.

Culture collection strain number	n-hexadecane	mineral oil
**EXF-2481**	-	-
**EXF-4632**	-	-
**EXF-12298**	-	-
**EXF-150**	-	-
**EXF-3670**	+	+
**EXF-10629**	-	-
**EXF-11318**	-	-
**EXF-5629**	-	-
**EXF-3398**	+	+

Legend: + assimilation, - no assimilation.

Table S3: Assimilation of neurotransmitters (acetylcholine – ACh, γ-aminobutyric acid – GABA, glycine – Gly, glutamate – Glu, dopamine – DA) of selected *Aureobasidium* species at 24 °C and 37 °C.

	**24 °C**					**37 °C**				
**Culture collection strain number**	**Ach**	**GABA**	**Gly**	**Glu**	**DA**	**Ach**	**GABA**	**Gly**	**Glu**	**DA**
**EXF-2481**	+	+	-	+	+	NG	NG	NG	NG	NG
**EXF-4632**	+	-	-	+	NG	NG	NG	NG	NG	NG
**EXF-12298**	+	+	+	+	=	NG	NG	NG	NG	NG
**EXF-150**	+	+	=	+	=	NG	NG	NG	NG	NG
**EXF-3670**	+	+	=	+	+	NG	NG	NG	NG	NG
**EXF-10629**	+	+	=	=	NG	NG	NG	NG	NG	NG
**EXF-11318**	+	+	=	=	=	NG	NG	NG	NG	NG
**EXF-5629**	+	-	-	-	NG	NG	NG	NG	NG	NG
**EXF-3398**	+	+		+	+	NG	NG	NG	NG	NG

Legend: + better growth, = similar growth, - worse growth, NG – no growth.

Table S4: Found non-ribosomal protein synthase (NRPS) genes with adenylation domains (A domains) similar to those of genes encoding proteins SidC and SidD in *A. melanogenum* genomes.

**Present study number**	**SidC**	**SidD**	**both**
**1**	1	1	2
**2**	1	2	3
**3**	1	1	2
**4**	1	1	2
**5**	1	1	2
**6**	2	2	4
**7**	1	1	2
**8**	2	2	4
**9**	3	2	5
**10**	2	2	4
**11**	1	1	2
**12**	1	1	2
**13**	1	1	2
**14**	1	1	2
**15**	1	1	2
**16**	3	2	5
**17**	3	2	5
**18**	1	1	2
**19**	2	2	4
**20**	3	2	5
**21**	3	2	5
**22**	3	2	5
**23**	3	2	5
**24**	3	2	5
**25**	1	1	2
**26**	2	2	4
**27**	1	1	2
**28**	2	1	3
**29**	2	2	4
**30**	2	2	4
**31**	3	2	5
**32**	2	1	3
**33**	3	2	5
**34**	2	2	4
**35**	1	2	3
**36**	2	2	4
**37**	3	2	5
**38**	1	2	3
**39**	2	2	4
**40**	1	1	2
**41**	2	2	4
**42**	2	2	4

**43**	3	2	5
**44**	3	2	5
**45**	3	2	5
**46**	3	2	5
**47**	1	2	3
**48**	1	1	2
**49**	3	2	5

Table S5: Found hemolysins homologues from three different groups in *A. melanogenum* genome.

Present study number	First group	Second group	Third group
**1**	1	1	2
**2**	0	1	2
**3**	1	1	2
**4**	1	1	2
**5**	1	1	2
**6**	2	2	6
**7**	1	1	2
**8**	2	2	3
**9**	1	2	4
**10**	1	2	4
**11**	1	1	2
**12**	1	1	2
**13**	1	1	2
**14**	1	1	2
**15**	1	1	2
**16**	0	2	4
**17**	1	1	4
**18**	1	1	2
**19**	1	2	4
**20**	2	1	4
**21**	2	2	4
**22**	2	2	4
**23**	2	2	4
**24**	1	1	4
**25**	1	1	2
**26**	1	3	4
**27**	1	1	2
**28**	1	1	4
**29**	2	1	4
**30**	2	1	4
**31**	2	2	4
**32**	2	1	4
**33**	2	2	4

**34**	1	2	4
**35**	0	1	1
**36**	2	2	6
**37**	1	2	4
**38**	0	1	2
**39**	1	2	4
**40**	1	1	2
**41**	2	2	6
**42**	2	1	4
**43**	1	2	4
**44**	1	3	4
**45**	1	2	4
**46**	1	2	4
**47**	1	1	2
**48**	1	1	2
**49**	1	2	4

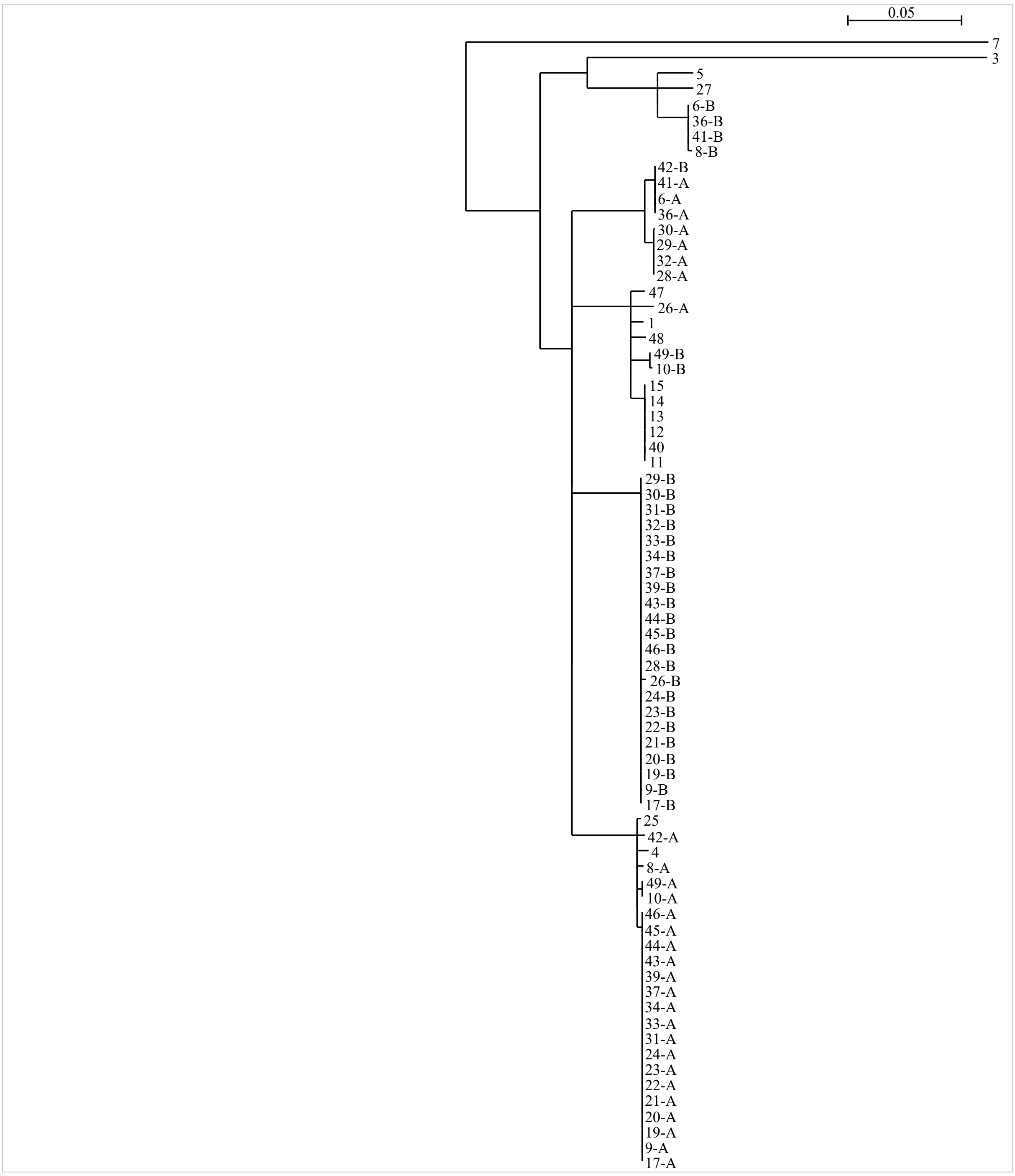

Figure S1: Majority rule consensus tree of *A. melanogenum* Benchmarking Universal Single-Copy Orthologs (BUSCOs). Strains 2, 16, 18, and 38 were excluded from the analysis due to a large share of their BUSCOs predicted as single-copy instead of in the expected two copies and strain 35 was excluded due to its large phylogenetic distance to other strains.

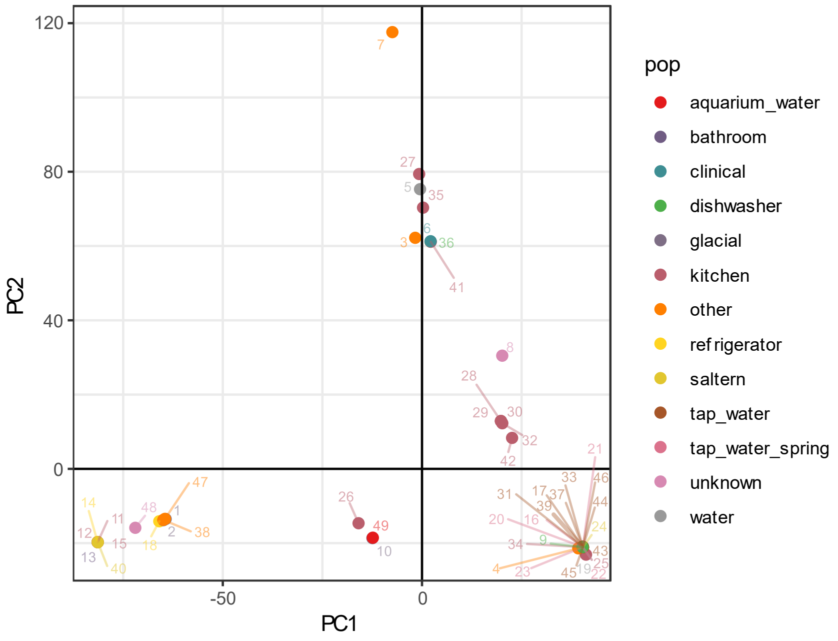

Figure S3: Clustering of *A. melanogenum* genomes. Principal component analysis of SNP data estimated by comparing the genomes to the reference *A. melanogenum* genome (EXF-3378). The genomes are represented by circles, the colour corresponds to the habitat. The axes explain 33,11% (horizontal) and 19,85% (vertical) of variation.

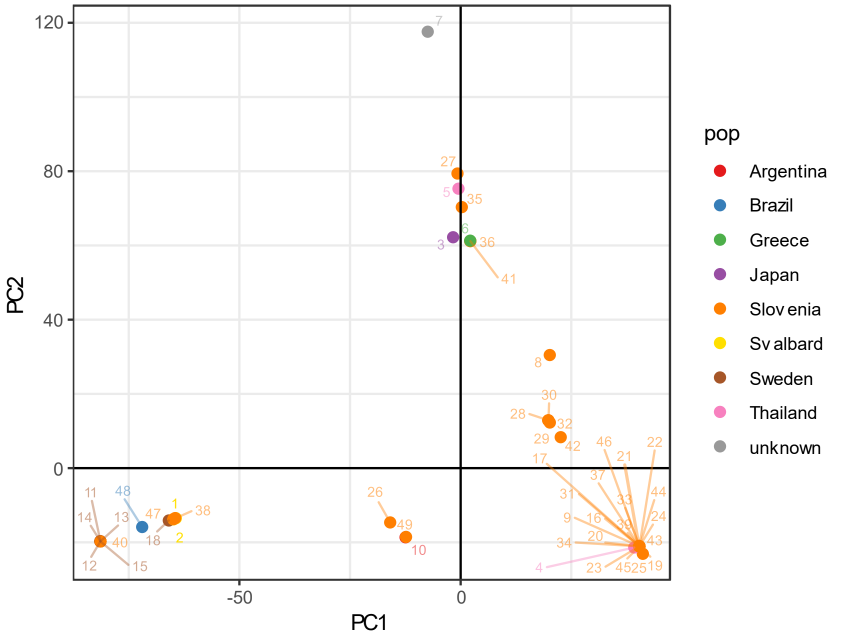

Figure S4: Clustering of *A. melanogenum* genomes. Principal component analysis of SNP data estimated by comparing the genomes to the reference *A. melanogenum* genome (EXF-3378). The genomes are represented by circles, the colour corresponds to the sampling location. The axes explain 33,11% (horizontal) and 19,85% (vertical) of variation.

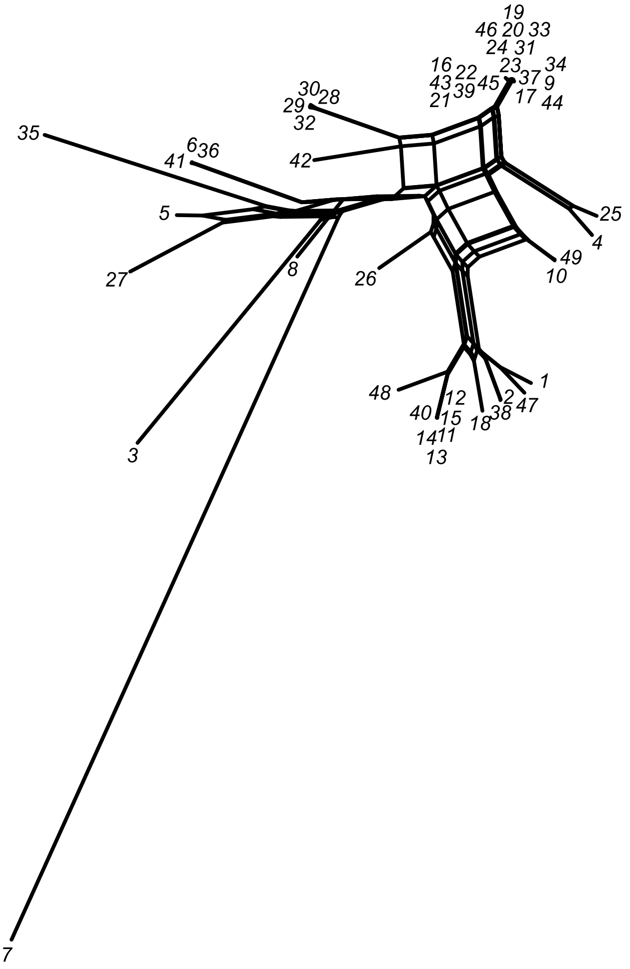

Figure S2: Phylogenetic network of sequenced *A. melanogenum* strains, reconstructed with the Neighbor-Net algorithm based on the dissimilarity distance matrix calculated from the SNP data.
